# Effect of Temperature and Strain Rate on the Flexural Behavior of Wood-Polypropylene Composites

**DOI:** 10.3390/ma12233987

**Published:** 2019-12-02

**Authors:** Wei Wang, Xiaomin Guo, Liu Liu, Ruiyun Zhang, Jianyong Yu

**Affiliations:** 1 Key Laboratory of Textile Science Technology, Ministry of Education, College of Textiles, Donghua University, Shanghai 201620, China; 1152020@mail.dhu.edu.cn; 2 Shandong Academy of Sciences Institute of Information, Qilu University of Technology (Shandong Academy of Sciences), Jinan 250014, China; gxmstc@163.com; 3 Innovation Center for Textile Science and Technology, Donghua University, Shanghai 201620, China; llisatc@126.com (L.L.); yujy@dhu.edu.cn (J.Y.)

**Keywords:** wood powder, polypropylene, injection technique, time-temperature superposition principle

## Abstract

The mechanical properties of wood-polypropylene composites exhibit typical viscoelasticity. However, there is little information on the mechanical properties of wood-polypropylene composites related to temperature and time, which limits the use of wood-polypropylene composites as structural components. Here, the effect of time (strain rate) and temperature on the flexural properties and the master curve of the storage modulus used to predict the long-term performance of wood-polypropylene composites were investigated. The results showed that the flexural strength and modulus increased linearly with the increase of wood contend, which can increase by 134% and 257% respectively when the mass fraction of wood powder reached 45%. Moreover, there was a positive linear relationship between flexural strength and ln strain rate, while the flexural strength and modulus decreased as temperature elevated. The storage modulus as a function of frequency (time) and temperature confirmed this trend. To evaluate the long-term performance, the storage modulus master curve was constructed and the respective activation energy was calculated, which revealed that the long-term performance of the samples depended on the matrix and the addition of an appropriate amount of wood powder was beneficial to improve their durability.

## 1. Introduction

In the past few decades, extensive research has been reported on composites reinforced with natural materials (i.e., natural fibers, particles, etc.) due to their environmentally friendly properties [1,2,3,4,5]. Wood-plastic composite materials, as a kind of eco-friendly materials, combine the excellent performance of both wood and plastic. They have a great number of merits such as low cost, green environmental protection, better thermal and acoustic isolation, better durability, anti-corrosion, anti-mildew, recyclability and other engineering features [6,7,8]. They can replace plastics and wood on many occasions. Moreover, they have better strength and rigidity to replace traditional high-performance fiber or glass fiber reinforced composites, thus helping to alleviate increasingly serious environmental problems and resource shortages [9,10,11].

Wood-plastic composites have attracted great consideration in the fields of automobiles, architecture, furniture and household articles [9]. In the academic field, the research of wood-plastic materials is mainly focused on the production formula and production process [12,13,14,15], as well as the mechanical properties [16,17,18]. The common methods of the preparation of wood-plastic composites include extrusion, hot press molding, injection molding and so forth. Some researchers specialized in the process parameters in the molding process of wood-plastic materials to improve the mechanical properties of wood-plastic products by adjusting the process parameters including the rotation speed and advance speed of the screw, the shape and length of the screw and the temperature in the different molding processes [12]. Some researchers focused on improving the interfacial bonding of wood-plastic materials [19,20,21]. The nature of the interface is an important factor influencing the properties of the wood-plastic composite but there is a compatibility problem between the hydrophilic surface of the wood powder and the hydrophobic surface of thermoplastic materials. Therefore, it is necessary to modify the surface of the wood powder or the resin or to introduce some compatibilizers in the mixing process to make the interface has proper adhesion and good dispersion. Besides, many researchers were also keen to study the functionalization of wood-plastic composites, for example, flame-retardant wood-plastic materials were prepared by mixing flame retardant agents with wood powder, polymer and other additives [22,23,24].

For any material, mechanical properties are its most basic performance indicators. The polymer is a typical viscoelastic material whose mechanical properties are influenced by time and temperature. Wood-plastic materials composed of the polymer matrix and wood materials also have viscoelastic characteristics and exhibit mechanical properties related to time and temperature in service. Wood-plastic composites have not been widely used in structural applications because their long-term performance has not been adequately studied and there is not enough performance data to support their rational engineering. However, it is not easily accessible experimentally to acquire the performance data with a very long range of timescale. Therefore, to find a way to accelerate trials to study the long-term performance of the material is very urgent matters.

Many researchers have used the principle of time-temperature equivalence to study the mechanical properties concerning time and temperature. If the temperature increases, the relaxation time will shorten and a certain predetermined performance index can be achieved in a relatively short time. On the contrary, if the temperature decreases, the relaxation time will be extended to achieve the predetermined index of the same effect, where the temperature is used as the accelerant. The time-temperature superposition principle (TTSP) is a useful and practical application of the principle of time-temperature equivalence, which can be used to study and predict the long-term performance of materials [25,26,27,28]. Moreover, there are some restrictions when applying the TTSP. At first, the use of the TTSP is applied to amorphous materials and then the researchers further extended the scope of their application. If the deformation of the semi-crystalline material (such as polypropylene) used herein is in the range of linear viscoelasticity, its long-term durability can be studied by the TTSP [28,29,30]. Furthermore, the activation energy of the polymer materials can be calculated according to the shift factor in the TTSP and which can be used to evaluate the thermal stability and the durability of materials [31,32,33].

Although researchers have achieved a lot of results in the long-term performance of materials using the TTSP, there are few studies on the long-term performance of wood-polypropylene composites and there is not enough performance data support for their application as structural parts.

This paper is concerned with the effect of strain rate and temperature on the static flexural properties and the effect of frequency and temperature on the dynamical mechanical performance, as well as the prediction of long-term performance of pure polypropylene (PP) and wood powder (WP)-filled polypropylene composites. PP and PP/WP composites with different wood powder content were prepared by injection technique. The three-point bending tests of the samples were conducted under the conditions of various temperatures and strain rates. Furthermore, the scanning electron microscope (SEM) was used to characterize the fractured surface of the samples and the dynamic mechanical analysis (DMA) was applied to testify the time-temperature dependence of PP and PP/WP composite samples. The characteristics of time and temperature dependence of flexural behavior for the samples were discussed. Eyring equation and Arrhenius relationship were employed to analyze the activation volume and activation energy of PP and PP/WP composite samples, respectively. Moreover, the master curve of the storage modulus was constructed following the TTSP.

## 2. Materials and Methods

### 2.1. Materials

Polypropylene (PP) pellets for injection molding with a melt mass-flow rate of 3 g/10 min were prepared by Prime Polymer Co., Ltd., Osaka, Japan and PP/WP granules containing 70 wt% wood powder were made by Plesir Co., Ltd., Osaka, Japan. In addition, the maleated polypropylene (MAPP) was a compatibilizer for improving the interfacial properties of wood powder and polypropylene.

### 2.2. The Preparation of WP/PP Composite Samples

The sample for the three-point bending test was injection-molded from PP pellets containing 70 wt% wood powder and pure PP pellets. All raw materials were dried in a hot-air oven for 12 h to remove moisture from the materials ahead of the injection molding. Two kinds of pellets uniformly mixed in a certain mass ratio were fed into the injection molding machine and the rotation speed of the screw was 185 rpm and the forward speed was 30 mm/s. The barrel temperature of the injection molding machine was 160–170 °C and the molding temperature was 30 °C PP/WP composites with different wood powder content were obtained by adjusting the mass ratio of PP/WP granules containing 70% wood powder and pristine PP pellets. The prepared samples were designated as WP0 (pure PP), WP15 (with 15 wt% wood powder), WP30 (with 30 wt% wood powder) and WP45 (with 45 wt% wood powder).

### 2.3. Three-Point Bending Tests

Three-point bending tests were conducted using an Instron tester (Instron, Norwood, Massachusetts, USA) with a hot-air oven that can provide a range of temperatures from room temperature to 100 °C. The span of the base unit in the three-point bending test was set at 48 mm with a span-to-depth ratio of 16:1. The load-deflection curves were recorded by the machine during the deformation of the samples and the stress-strain curves of samples can be calculated accordingly. The loading speeds employed at each set temperature were 0.2, 2 and 20 mm/min and the corresponding strain rates were 2.6 × 10^−3^, 2.6 × 10^−2^ and 2.6 × 10^−1^ s^−1^ respectively. The values of flexural strength, flexural modulus and flexural strain were calculated according to ISO 178-2010 and each index was obtained by the mean values of three specimens tested under the same conditions. The fractured surfaces of WP45 tested under different temperatures were observed by a scanning electron microscopy (JEOL-Model 6309, JEOL Ltd., Akishima, Japan).

### 2.4. Dynamic Mechanical Analysis (DMA)

Dynamic mechanical analysis of the samples with standard size (length, width and thickness of 60 mm, 10 mm and 3 mm, respectively) was carried out using a dynamic mechanical analyzer (DMA 2980, TA Instruments, New Castle, Delaware, USA). The tests employed a multi-frequency-dual cantilever mode and the testing conditions were controlled in a heating rate of 3 °C/min and the amplitude of 15 μm, with the temperature range from −60 °C to 100 °C at intervals of 20 °C and the frequencies viz 0.2, 2, 20, 200 Hz for each temperature step.

## 3. Results and Discussion

### 3.1. Effect of Wood Content on the Flexural Behavior of the Samples

**Figure 1:**
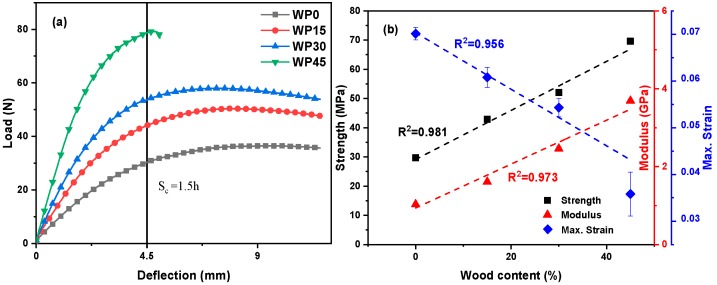
Flexural behavior: (**a**) load–deflection curves of PP and PP/WP composite samples at 25 °C; (**b**) the relationship between the flexural behavior and wood content of specimens.

Figure 1a delineates the representative load-deflection behavior of pure PP and PP/WP composite samples. It is found that the maximum load of the samples of WP0, WP15, WP30 and WP45 are notably different and the maximum load increases with the increase of wood powder content, which shows a great reinforcing effect of wood powder on PP matrix. Furthermore, both flexural strength and flexural modulus of the samples increase linearly with the increase of wood content as shown in Figure 1b, the flexural strength increases from 29.69 MPa for WP0 to 42.91 MPa, 52.03 MPa and 69.61 MPa for WP15, WP30 and WP45 respectively, which increases 44.5%, 75.2% and 134.5%. Similarly, the flexural modulus also improves from 1033 MPa for WP0 to 1610 MPa, 2463 MPa and 3690 MPa for WP15, WP30 and WP45 respectively, which increases 55.9%, 138.4% and 257.2%. However, the deflection at the maximum load decreases as wood powder content increases. Specifically, as shown in Figure 1b, the inverse relationship between the maximum strain (the strain at the maximum load) and wood powder content is observed and the maximum strain of WP45 is nearly half that of pure PP, which indicates that the existence of wood powder reduces the toughness of PP matrix. The curve of sample WP45 is distinguished from the curves of other samples according to the given deflection (S_c_ = 1.5 h) marked in Figure 1a. The load-deflection curves of samples WP0, WP15 and WP30 remain to level off after reaching the maximum load point and then slowly decrease. This indicates that the sample does not break at the maximum stress point and the fracture is a ductile failure. On the other hand, when the wood powder content is high (WP45), the load-deflection curve of the sample reaches the highest load point and then drops sharply to a load of zero. This shows that the material does not yield and directly breaks, the maximum stress (flexural strength) is equal to the fracture flexural strength and the fracture is brittle. As a result, the addition of wood powder enhances the matrix’s resistance to misalignment and the lignin and wood fibers in wood powder provide high strength and stiffness to the composite while destroying the continuity of the matrix and reducing the toughness of the material, which corresponds to the previously reported results [34]. As the wood powder content increases, the failure mode of the composites is converted from ductile failure to brittle failure.

### 3.2. Effect of Strain Rate on the Flexural Behavior of the Samples

**Figure 2:**
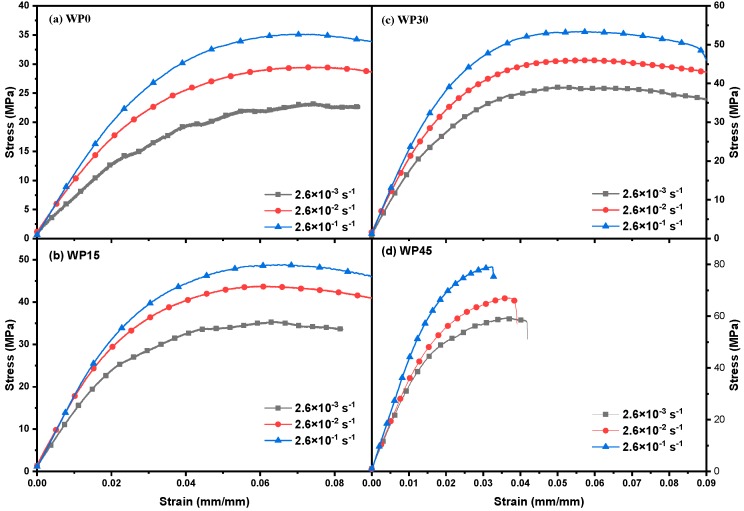
Stress–strain curves of (**a**) WP0, (**b**) WP15, (**c**) WP30 and (**d**) WP45 at 25 °C for various strain rates.

Typical bending stress-strain plots of the samples tested at 25 °C with different strain rates are presented in Figure 2, which shows the transition of the sample from elastic deformation to plastic deformation or fracture. Firstly, the elastic deformation occurs due to the change of the bond lengths and bond angles of macromolecules and the magnitude of the deformation is proportional to the magnitude of the external force, that is, the stress-strain relationship is linear and obeys Hooke’s law. When the external force is increased to a certain extent, some transverse joint bonds are broken, so that the segments can move freely to produce large deformation without increasing the stress [35,36]. The curves show that the flexural strength under various strain rates was obviously different, that is, the value of the flexural strength increases with the increase of the strain rate while the value of the maximum strain decreases slightly. The fracture process of the material is essentially a relaxation process of the polymer segment and increasing the strain rate means shortening the relaxation time.

**Figure 3:**
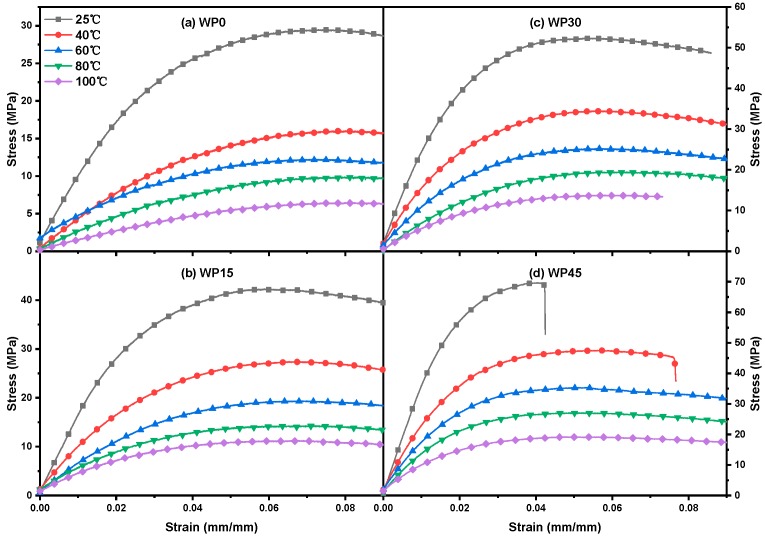
Stress–strain curves of (**a**) WP0, (**b**) WP15, (**c**) WP30 and (**d**) WP45 at a strain rate of 2.6 × 10^−2^ s^−1^ for various temperatures.

Figure 3 indicates the plots of stress versus strain for WP0, WP15, WP30 and WP45 with a strain rate of 2.6 × 10^−2^ s^−1^ under different temperatures. It is found that the flexural strength of PP and PP/WP composites varies with the testing temperature. Referring to Figure 2 and Figure 3, it can be known that the bending properties of PP and PP/WP composites are dependent on the test temperature and the strain rate of the material, to be more specific, the flexural strength increases with the increase of strain rate and decreases with increasing temperature. Moreover, the flexural modulus shows a similar trend as the flexural strength, which can be observed by the slope of the tangent of the initial segment of the stress-strain curve (Figure 2 and Figure 3). The bending behavior can be thought of as a relaxation process caused by the motion of the segment. The relationship between relaxation time and temperature is in line with Eyring’s general theory of velocity processes. Therefore, Eyring equation are used to delineate the relationship between flexural strength and strain rate as well as temperature as follows [37]:

(1) $$\frac{\sigma }{T}\;=\;\frac{\Delta H}{vT}\;+\;\frac{k}{v}\;\ln\;\left(\frac{2\varepsilon^{\prime }}{\varepsilon_{0}^{\prime }}\right)$$

where σ is the flexural strength, T is absolute temperature, ΔH is the plastic deformation activation energy, that is, the energy required for the activation of the corresponding motion unit, v is the activation volume, k is Boltzmann constant, $\varepsilon^{\prime }$ is the strain rate and $\varepsilon_{0}^{\prime }$ is the pre-exponential factor. From Equation (1), it can be concluded that the bending strength σ has a linear relationship with ln ($\varepsilon^{\prime }$) and the activation volume of the material can be calculated from the slope (*s*) of the straight line.

(2) $$v=\frac{kT}{s}$$

**Figure 4:**
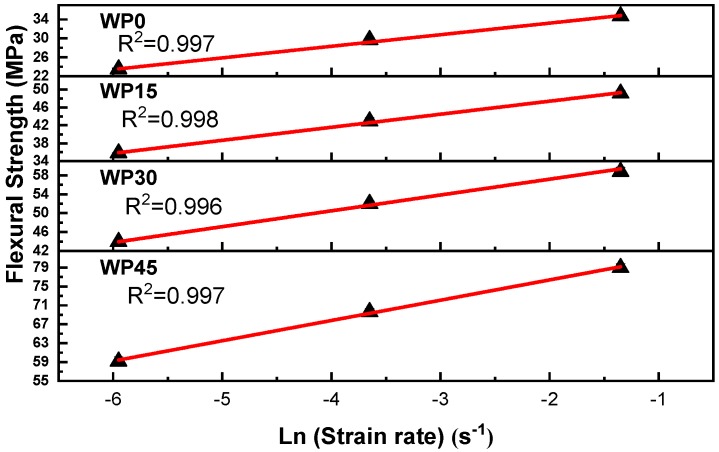
Strain rate dependence of flexural strength at 25 °C. The straight lines are fitted by linear regression method and R^2^ is the coefficient of regression.

Figure 4 delineates the relationship of flexural strength and strain rate for pure PP and PP/WP composites tested at 25 °C. Table 1 presents the slope of regression lines and activation volume for pure PP and PP/WP composites. As shown, the addition of wood powder into the PP matrix resulted in a reduction in the activation volume of the composites. It is reported that the coordinated movement of molecular segments forms the plastic flow of the materials [37]. The activation volume of composites showed a downward trend with the increase of wood powder content. The wood powder content becomes higher and in contrast, the polypropylene content becomes less. On the other hand, the movement of PP molecular chain segments was considered to be limited by wood powder. Therefore, the higher the content of wood powder, the greater the resistance to the movement of polypropylene molecular segments and the smaller the activation volume in the materials.

### 3.3. Effect of Temperature on the Flexural Behavior of Samples

**Figure 5:**
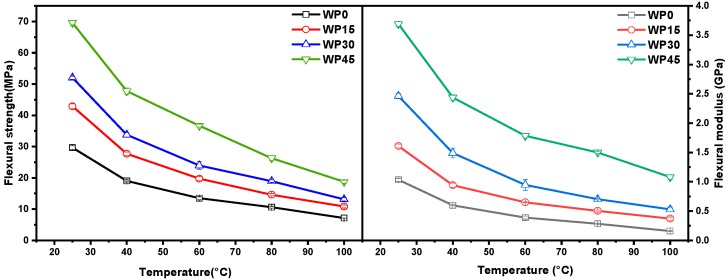
Flexural stress and modulus vs. temperature plots at a strain rate of 2.6 × 10^−2^ s^−1^.

**Figure 6:**
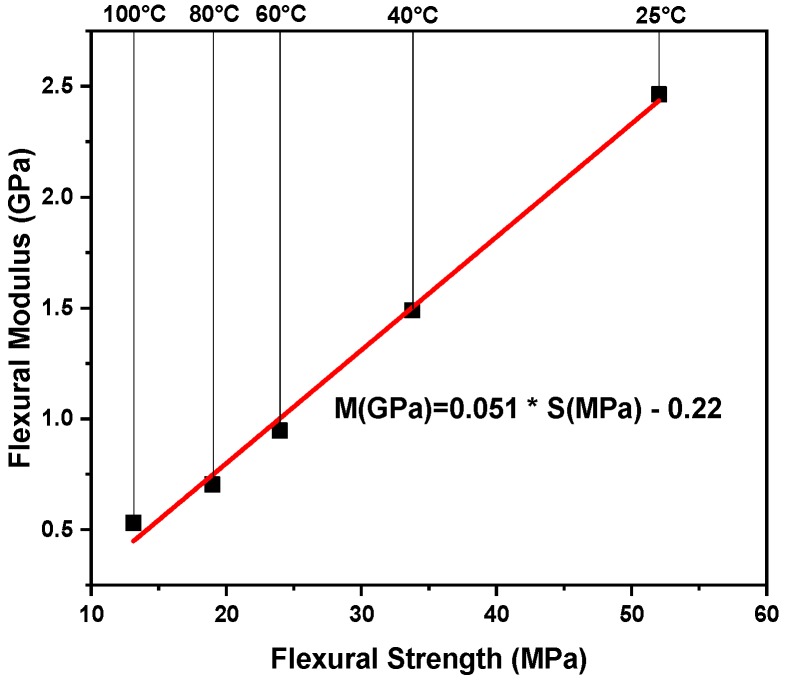
Flexural modulus vs. strength for WP30.

A comparatively low strain rate (2.6 × 10^−2^ s^−1^) was selected to study the effect of temperature on the flexural properties of the samples. As discussed in the previous section, the temperature affects both flexural strength and flexural modulus of PP and PP/WP composites. More specially, flexural strength and modulus versus temperature plots for pure PP and PP/WP composites at the chosen strain rate are presented in Figure 5, both flexural strength and modulus decrease with increasing the test temperatures. Furthermore, for example, the plot of flexural modulus versus the flexural at various temperatures for sample WP30 is shown in Figure 6, it can be found that there is a linear relationship between the strength and the modulus, which means that they maintain a proportional reduction as the test temperatures increase. Therefore, the variation of flexural modulus with time can reflect the variation of flexural strength with time.

**Figure 7:**
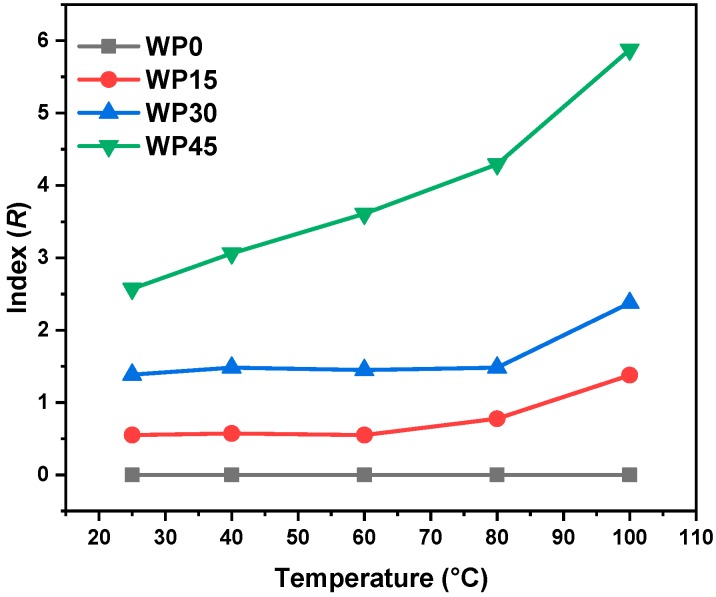
Plots of index (*R*) as a function of temperature.

**Figure 8:**
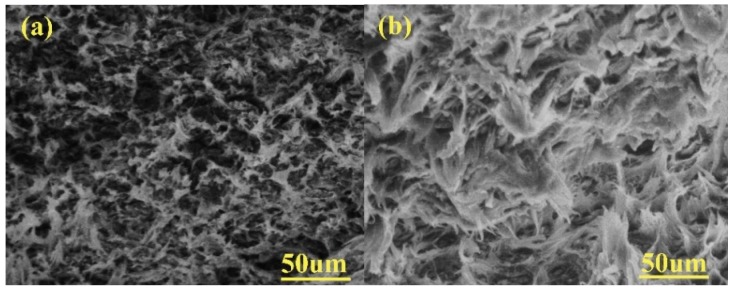
Scanning electron microscope (SEM) fractographs (×350) of WP45 tested at temperature of (**a**) 25 °C and (**b**) 80 °C.

To study whether the enhancement effect of wood powder on PP matrix is the same at different test temperatures, the enhancement index is used to characterize the enhancement effect of wood powder on PP matrix at each temperature and the enhancement index (*R*) is defined as follows:

(3) $$R=\frac{M^{T}-M_{0}^{T}}{M_{0}^{T}},$$

where $M^{T}$ and $M_{0}^{T}$ represent the flexural modulus of PP/WP composites and pure PP at a certain temperature *T*, respectively. It can be found that the index “*R*” is positively related to the flexural modulus $M^{T}$ and the higher the value of “*R*,” the higher the flexural modulus. In other words, this index is positively correlated with the enhancement effect of wood powder on the PP matrix and the large index indicates that the enhancement effect is great. First, at any temperature (25–100 °C), the enhancement effect of PP/WP composites is ranked as follows—WP45 > WP30 > WP15. More importantly, the enhancement effect improves with the elevated temperature, especially for the sample with high wood powder content (WP45). These results indicate that the enhancement effect of wood powder on the PP matrix is related to its content and test temperature. It can be seen from Figure 7 that the enhancement effect is more significant at higher temperatures. Figure 8 includes two SEM images of the fracture morphology of WP45 tested at 25 °C and 80 °C, respectively. The fracture surface is transformed from the brittle mode into the ductile mode with increasing testing temperatures. The wood powder can bridge the microcracks of the PP matrix effectively during deformation and the molecular segments move faster when the test temperature is high. Thus, the more wood powder and the higher the temperature, the more significant the effect of bridging the microcracks of PP.

### 3.4. Application of Time-Temperature Superposition Principle to the Composite

**Figure 9:**
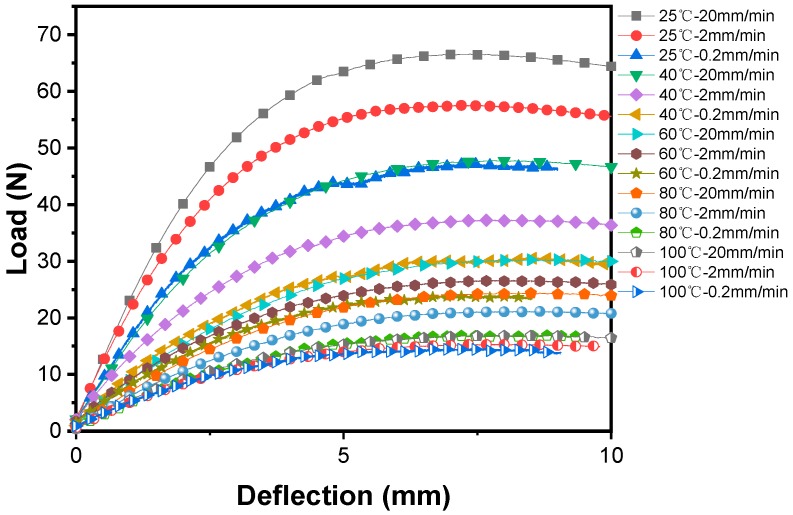
Load-deflection curves of WP30 at various temperatures and loading rates (strain rates).

As mentioned above, the flexural behavior of both pure PP and PP/WP composites is related to temperature and strain rate. More importantly, the effect of increasing temperature is equivalent to that of lowing the strain rate (prolong time). In other words, the mechanical response at low temperature is equal to the response in a short time and the response at high temperature is equal to the response in a long time [38], which can be confirmed by the results of the three-point bending tests as shown in Figure 9 taking sample WP30 as an example, in which the load-deflection curves at low temperatures and low strain rates (loading rates) can overlap the load-deflection curves at high temperatures and high strain rates.

**Figure 10:**
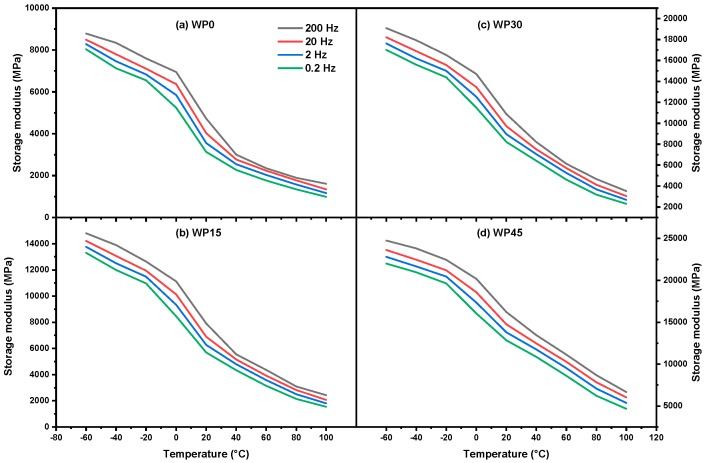
Storage modulus vs. temperature for (**a**) WP0, (**b**) WP15, (**c**) WP30 and (**d**) WP45.

Earlier studies have confirmed that the time-temperature superposition principle (TTSP) is valid for crystalline and semi-crystalline materials when the deformation is kept in the linear viscoelastic range [28,39]. DMA experiments are carried out with a very small strain, so the response of the material is in the linear viscoelastic range when employing the DMA test [38]. On the other hand, it is possible to observe a wider range of viscoelastic behavior with a temperature scan in the DMA test. The storage modulus in dynamic mechanical analysis of pure PP and PP/WP composites is a function of time and frequency as shown in Figure 10. As it is shown that the storage modulus increases with increasing frequency but decreases as temperature increases, which corresponds to the earlier reports [40,41,42], as well as the results obtained by three-point bending tests. In essence, the change of the frequency means the variation of the response (strain rate) of the material. Therefore, the master curve of the storage modulus can be constructed in accordance with the time-temperature superposition principle.

The frequency (*f*) in the DMA test is related to the experimental time *t* and the quantitative relation is as follows:

*t* = 1/2π*f*. (4)

**Figure 11:**
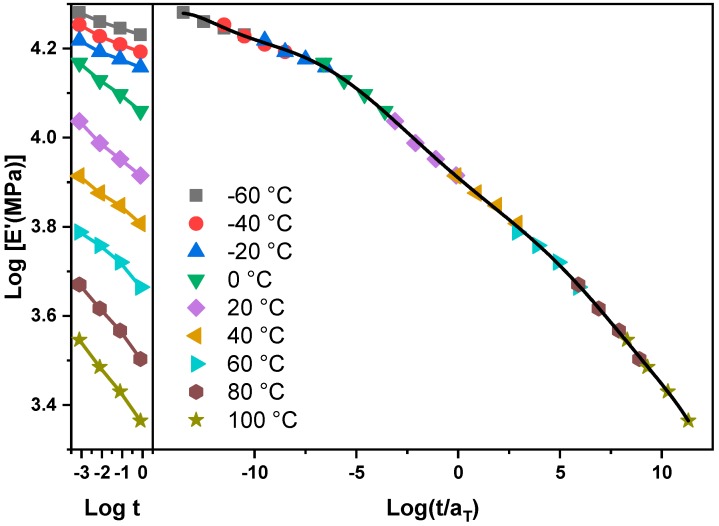
Log E’ vs. log t plot and master curve for WP30.

The storage modulus-time curve measured at a certain temperature can be superimposed on the storage modulus-time curve measured at various temperatures. Taking the WP30 sample as an example, the log E’ versus log t curves with a temperature range from −60 °C to 100 °C are shown on the left of Figure 11. It can be found that by shifting along the time axis, these curves can be superimposed together to form a very long storage modulus-time curve as shown on the right of Figure 11. More specifically, a plot of storage modulus versus time at a certain temperature is chosen as the reference and the corresponding temperature and time can be regarded as the reference temperature, $T_{R}$ ($T_{R}$ is 20 °C in the current case) and reference time $t_{R}$. Besides, the temperature and time corresponding to the curves shifted along the time axis can be marked as temperature *T* and time *t*. The distance along the logarithmic time axis, that is, shift factor, log *a_T_*, presents the difference of the relaxation rates between the temperature *T* and the reference temperature $T_{R}$.

**Figure 12:**
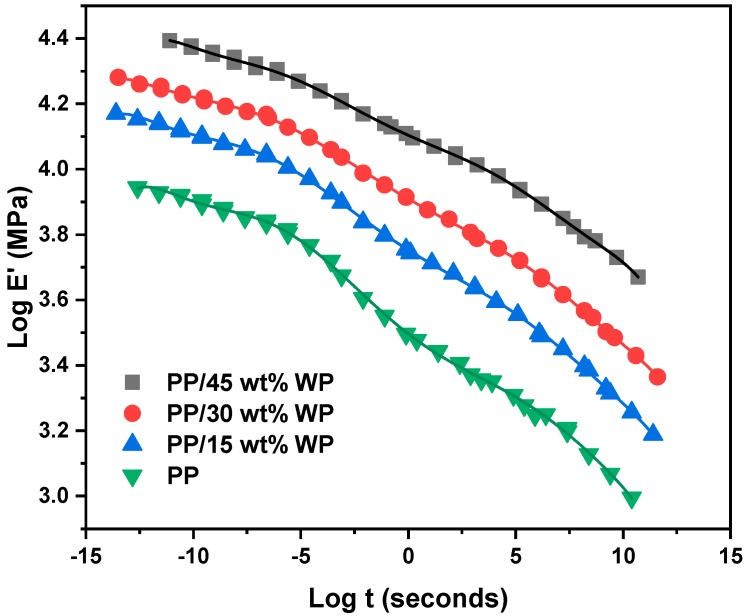
Time-temperature superposition principle master curves for PP and PP/WP composites.

The master curves of the storage modulus as a function of time for all samples (WP0, WP15, WP30 and WP45) are constructed according to shift factors and the TTSP (shown in Figure 12), which can be used to forecast the long-term mechanical behavior of materials and the mechanical performance under some conditions that the laboratory cannot achieve. As shown, the curves of WP15, WP30 and WP45 show a similar shape to WP0 and they are all similar to the plot of the storage modulus versus temperature, which confirmed that the equivalent of time and temperature. At first, the storage modulus decreases slowly along time and then drops quickly over a period of time, after that, the storage modulus reduces at a lower rate. It is noted that the value of the storage modulus of WP45 remains the highest in the time range and WP30, WP15, WP0 followed in order, which is because of the addition of wood powder. And as previously concluded, the reinforcement effect of wood powder is much more significant when the wood powder content and testing temperature are high. Moreover, the predicted time span of sample performance obtained in the same temperature range is different and the performance prediction time span of WP15 and WP30 is longer than that of WP0 and WP45. In other words, the time span reflects the time required for the mechanical properties of the material at −60 °C to transform to mechanical properties at 100 °C. It can be concluded that the long-term properties of PP/WP composite sample are based on the matrix, PP, though the addition of wood powder enhanced the mechanical properties of resulting materials, which can be confirmed by the previous study obtained the similar conclusion [43].

In summary, from the shape of the master curves of the storage modulus vs time, on one hand, it confirms the equivalence of time and temperature, on the other hand, it can be known that the long-term performance of the polymeric composite is dependent on the polymer matrix.

### 3.5. Evaluation of the Activation Energy for the Samples

The activation energy is used to define the energy barriers that need to be overcome to occur in a chemical reaction. The activation energy can be used to indicate the minimum energy required for a chemical reaction to occur. It is proved that any change in vibrational frequency may result in the change of glass transition temperature [32]. The change in glass transition temperature allows the calculation of the activation energy of each sample in the relaxation process according to the following Arrhenius equation:

(5) $$\log\;a_{T}=\;\log\frac{t}{t_{R}}\;=\;-\frac{\Delta H}{2.303R}\left[\frac{1}{T}-\frac{1}{T_{R}}\right]$$

where *R* is a gas constant, Δ*H* is the activation energy, *T* and $T_{R}$ are measuring temperature and reference temperature, respectively.

**Figure 13:**
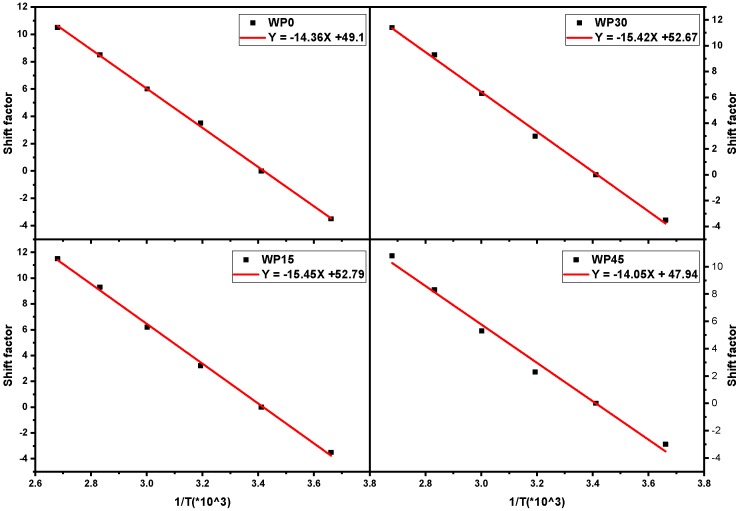
Shift factor for PP and PP/WP composites.

From Equation (5), the activation energy of the samples is proportional to the slope of log *a_T_* versus 1/*T* plot when the testing temperature is above the glass transition temperature as shown in Figure 13 (temperature range from 0 to 100 °C) and the magnitude of the activation energy of the sample can be calculated according to the slope of regression line. It can be seen that the activation energy of WP15 and WP30 is close, which is higher than that of WP0 and WP45. The similar results were obtained for other natural fiber reinforced polymer composites [33,44,45]. The specific values of the activation energy of the samples are listed in Table 1. Above the glass transition temperature, the free volume begins to thaw and the chain segment motion is excited. The samples with wood powder need to be provided with more energy because the movement of the molecular segments of the polypropylene needs to overcome the barrier of wood powder or the polypropylene molecular segments require more energy to move in concert with the wood powder. However, too much wood powder can easily lead to the accumulation of wood powder and the probability of stress concentration or defects of the aggregate particles is greatly increased, which affects the stress transfer between polypropylene and wood powder and the free movement of the chain segment [32]. From the standpoint of the respective activation energies of the samples, the durability of WP15 and WP30 is better because it takes longer for them to reach the mechanical behavior of 100 °C. On the other hand, in view of the specific values of mechanical properties, the rank of the specific value of the storage modulus is WP45 > WP30 > WP15 > WP0 in the test time range, which shows that sample WP45 is better. Therefore, activation energy is not the only indicator for assessing the long-term durability of materials. In addition, specific mechanical properties of materials and requirements for use conditions should also be considered.

## 4. Conclusions

Static flexural properties of pure PP and PP/WP composites were studied in terms of strain rate (time) and temperature, as well as wood powder content. The incorporation of wood powder was successful in increasing the flexural strength and flexural modulus of the composites and values of flexural strength and modulus increase linearly with the wood powder content. On the other hand, the flexural modulus and strength of the samples are temperature and strain rate dependent and the values of flexural strength and modulus improve with the increase of strain rate while they decrease as temperature increases. More importantly, it was found that the flexural strength was linear with the strain rate and the reinforcement effect of wood powder was more significant at elevated temperatures with more mass percentage. 

Time-temperature superposition principle (TTSP) was used to establish the master curve of the storage modulus as a function of time in dynamic mechanical analysis to evaluate the effect of wood powder on the long-term behavior of the composites and predict the trend of mechanical properties with time. The results showed that the long-term performance of PP/WP composites is dependent on the matrix, PP. Moreover, the activation volume of the composites decreased with the incorporation of wood powder. In the view of the activation energy, samples WP15 and WP30 have better durability due to the better even distribution of wood powder and the synergistic movement of wood powder and polypropylene segments.

## Figures and Tables

**Table 1:** Activation volume and activation energy for PP and PP/WP composites.

Samples	Slope *k*T/*v* (MPa)	Activation Volume *v* (nm^3^)	Activation Energy (kJ/mol)
WP0	2.45	1.68	274.88
WP15	2.91	1.41	295.73
WP30	3.35	1.23	295.19
WP45	4.29	0.96	269.09
